# Comparative evaluation of oxidative stress biomarkers F2-isoprostanes and 8-OHdG in Parkinson’s disease and Type 2 Diabetes Mellitus: a systematic review and meta-analysis of human studies

**DOI:** 10.1080/07853890.2026.2654251

**Published:** 2026-04-20

**Authors:** Samwel Sylvester Msigwa, Maiko Charles Mkwambe, Qiu-Yan Yu, Shi-Guo Zhu, Jian-Hong Zhu, Xiong Zhang, Jian-Yong Wang

**Affiliations:** ^a^ Department of Neurology, Institute of Geriatric Neurology, The Second Affiliated Hospital and Yuying Children’s Hospital, Wenzhou Medical University, Wenzhou, Zhejiang, China; ^b^ Department of Psychiatry and Mental Health, School of Medicine and Dentistry, The University of Dodoma, Dodoma, Tanzania; ^c^ Department of Pediatrics, Zhongnan Hospital of Wuhan University, Wuhan, China; ^d^ Department of Epidemiology and Statistics, School of Public Health, Wenzhou Medical University, Wenzhou, Zhejiang, China; ^e^ Wenzhou Key Laboratory of Neurogenetics, Wenzhou, Zhejiang, China; ^f^ Institute of Nutrition and Diseases and Center for Research, School of Public Health, Wenzhou Medical University, Wenzhou, Zhejiang, China

**Keywords:** Oxidative stress, F2-isoprostanes, 8-OHdG, diabetes, Parkinson’s disease, comorbidity

## Abstract

**Background:**

Oxidative stress is central to type 2 diabetes mellitus (T2DM) and Parkinson’s disease (PD). However, the utility of biomarkers for lipid peroxidation (F2-isoprostanes) and DNA damage (8-OHdG) in the comorbidity of PD and T2DM remains unclear.

**Methods:**

We conducted a systematic review and meta-analysis of 54 unique studies of human subjects aged ≥ 50 years (*n* = 7,521: 3,522 with T2DM, 722 with PD, and 3,277 controls), measuring biomarkers in serum, plasma, or leukocytes. Mixed-effects models quantified standardized differences (Hedges’ g) across subgroups.

**Results:**

In T2DM, F2-isoprostanes (*g* = 1.60, 95% CI: 0.95–2.25) and 8-OHdG (*g* = 2.64, 95% CI: 2.13–3.14) were markedly elevated (*p* < 0.001). Stronger effects were observed in younger cohorts and serum/plasma samples, with complications like nephropathy exhibiting extreme oxidative stress (*g* = 5.24). In PD, 8-OHdG was moderately elevated (*g* = 0.78, 95% CI: 0.18–1.39; *p* = 0.011), particularly in randomized controlled trials and plasma samples, whereas F2-isoprostanes were not significantly elevated (*g* = 0.47, 95% CI: −0.43–1.38). High heterogeneity in T2DM (I^2^ > 90%) reflected methodological variability.

**Conclusion:**

Distinct profiles – both markers elevated in T2DM but only 8-OHdG in PD – underscore 8-OHdG’s potential in PD-T2DM comorbidity. Future research should focus on standardized assays, multi-compartmental or multi-modal sampling, and longitudinal studies to clarify mechanisms and therapeutic targets.

## 1. Introduction

Parkinson’s disease (PD), the second most common neurodegenerative disorder, is characterized by the progressive loss of dopaminergic neurons in the substantia nigra, leading to motor impairments and non-motor symptoms [1–3]. By contrast, type 2 diabetes mellitus (T2DM), a metabolic disorder affecting over 500 million people worldwide, is marked by chronic hyperglycemia, insulin resistance, and systemic inflammation, often resulting in complications like nephropathy and retinopathy [4,5]. Despite their distinct clinical profiles, both conditions share a central pathogenic mechanism: oxidative stress, characterized by an imbalance between the production of reactive oxygen species (ROS) and antioxidant defenses [6–8]. Epidemiological evidence increasingly points to a bidirectional link between PD and T2DM, driven by shared pathways such as insulin resistance, mitochondrial dysfunction, neuroinflammation, and redox imbalance [9–11]. While T2DM is strongly associated with an increased risk of PD [12–15], the reverse association – from PD to T2DM – shows correlation but lacks clear causation [6,16].

In PD, oxidative stress significantly exacerbates neuronal damage through mechanisms such as mitochondrial dysfunction, impaired autophagy, and increased neuroinflammation, with dopaminergic neurons being particularly vulnerable [7,17]. Conversely, in T2DM, hyperglycemia disrupts mitochondrial function, leading to an overproduction of ROS [18], which in turn causes lipid peroxidation, DNA (deoxyribonucleic acid) damage, and pro-inflammatory signaling [18,19]. Despite differences in their primary manifestations, both conditions exhibit considerable overlap in oxidative stress pathways, suggesting that redox imbalance may be a critical link between PD and T2DM [10,11,20]. However, it is important to note that much of the evidence supporting shared oxidative mechanisms is derived from studies examining PD and T2DM independently rather than from populations with confirmed co-occurrence of both conditions. Consequently, the extent to which these overlapping pathways reflect true comorbidity at the individual level remains insufficiently characterized, particularly in older adults, where both diseases are most prevalent.

Blood-based biomarkers provide critical insights into oxidative damage, with F2-isoprostanes and 8-hydroxy-2′-deoxyguanosine (8-OHdG) being two of the most rigorously validated indicators [21,22]. F2-isoprostanes, stable byproducts of arachidonic acid peroxidation, are widely regarded as the gold standard for assessing lipid peroxidation, a process closely tied to metabolic dysregulation in T2DM [23]. Similarly, blood-based 8-OHdG quantifies cumulative oxidative DNA damage, implicated in both diabetic complications and the neurodegenerative progression of PD [10,24,25]. Notably, a recent large‐scale cohort of 2858 adults aged 18–65 showed that activation of the three major physiological stress systems (immune‑inflammatory, hypothalamic–pituitary–adrenal [HPA]‑axis, and autonomic nervous system) is positively associated with both plasma 8‑OHdG and F_2_‑isoprostane levels in a dose‑response fashion [26]. Although extensive research has examined these biomarkers separately in T2DM [27,28] and PD [29–31], showing consistent elevations in T2DM and variable results in PD, studies directly comparing their levels in patients with both conditions are scarce. Thus, while these biomarkers are well validated within each disease, their ability to capture shared oxidative signatures relevant to confirmed PD–T2DM comorbidity remains largely unexplored. Moreover, the evidence base is limited by methodological inconsistencies that hinder a clear understanding of the shared oxidative mechanisms in PD and T2DM. Variability in assay techniques, such as enzyme-linked immunosorbent assays (ELISA), gas chromatography-mass spectrometry (GC-MS), and high-performance liquid chromatography with electrochemical detection (HPLC-ECD), combined with differences in sample types (e.g. serum versus plasma), has led to conflicting findings [11,32–34]. These discrepancies impede cross-study comparisons and prevent definitive conclusions about oxidative stress in PD-T2DM comorbidity. This underscores the need for a systematic approach, such as a meta-analysis, to standardize biomarker assessments and identify reliable indicators of shared pathophysiology, paving the way for improved early diagnostic and therapeutic strategies targeting oxidative damage.

To tackle these challenges, this systematic review and meta-analysis synthesizes human studies on blood-based F2-isoprostanes and 8-OHdG in PD and T2DM, focusing on participants aged 50 years and above, a group with an elevated prevalence of both conditions. Our objectives are to (1) quantify and compare biomarker levels across both diseases using standardized effect sizes while assessing how methodological factors, such as assay type and sample source, affect variability and (2) assess whether convergent patterns of biomarker alterations across these diseases support the hypothesis of shared oxidative mechanisms relevant to PD–T2DM comorbidity, while acknowledging the current scarcity of direct evidence from cohorts with confirmed co-occurrence.

## 2. Methods

### 2.1. Study design

The systematic review protocol was developed in accordance with the guidelines outlined in the Preferred Reporting Items for Systematic Reviews and Meta-Analyses (PRISMA) [35]. Before initiating data collection, the planned, systematic review and meta-analysis were registered with PROSPERO (Registration number: CRD42025633253).

### 2.2. Search strategy and eligibility criteria

A comprehensive electronic search was conducted across three databases – PubMed, Web of Science (WOS), and Scopus – to identify relevant human studies published up to December 20, 2024. The search terms encompassed both disease conditions and biomarkers. For participants, terms included ‘Parkinson’s Disease’, ‘Parkinson*’, ‘PD’, ‘Diabetes Mellitus’, ‘Diabetes’, and ‘DM’. For the intervention and outcomes, terms related to oxidative stress, mitochondrial dysfunction, and the biomarkers of interest were used, such as ‘F2-isoprostane’, ‘8-OHdG’, ‘8-iso-PGF2α’, ‘8-epi-PGF2α’, ‘15-F2t-isoprostane’, and ‘iPF2α-III’. Healthy control subjects were identified as ‘Control’ or ‘Healthy Control’. Boolean operators combined these terms, and the search was refined to exclude animal and *in vitro* studies. All retrieved references were exported to Covidence (Veritas Health Innovation, Melbourne, VIC, Australia) for deduplication and further screening. Duplicate records were initially removed using EndNote version X7 (Clarivate Analytics, Philadelphia, USA), followed by additional deduplication *via* Covidence. The eligibility criteria were as follows: Participants: Human subjects aged 50 years and above diagnosed with T2DM or PD, with control groups consisting of non-diseased individuals without significant comorbidities; Intervention(s): Measurement of blood-based F2-isoprostanes and/or 8-OHdG levels; Comparator(s): Healthy control subjects; Outcomes: Blood levels of F2-isoprostanes and 8-OHdG as markers of oxidative stress; Study Designs: Cross-sectional studies, case-control studies, cohort studies, and clinical trials; Exclusions: Non-English articles, reviews, editorials, studies without precise biomarker data for F2-isoprostanes or 8-OHdG, studies involving DM with infection (e.g. diabetic foot), and Type 1 Diabetes Mellitus (T1DM). Detailed search information is provided in the Supporting Information (Supplementary Tables S1–S3).

**Table 1.** Summary of studies on F2-isoprostanes in Diabetes Mellitus and Parkinson’s disease.

Author (Year)	Location	Study design	Sample size (DM/PD/Controls)	Mean age (Years)	Sample source	Assay type	Diagnostic criteria	Results (DM vs Control)	Results (PD vs Control)
Wang et al. (2024) [43]	USA	Cohort	132 / 0 / 571	DM: 73.9 ± 2.5, Controls: 73.6 ± 3.0	Plasma	GC-MS	Clinically diagnosed T2DM	F2-Isoprostanes: DM − Control	–
Kaliaperumal et al. (2022) [44]	India	Cross-sectional	60 / 0 / 60	DM: 59.18 ± 7.51, Controls: 62.57 ± 7.56	Serum	ELISA	Clinically diagnosed T2DM	F2-Isoprostanes: DM ↑ Control	–
Ma et al. (2021) [45]	China	Cross-sectional	66 / 0 / 53	DM: 58.33 ± 7.77, Controls: 55.42 ± 9.71	Plasma	ELISA	WHO criteria for T2DM	F2-Isoprostanes: DM ↑ Control	–
Morsi et al. (2018) [46]	KSA	Cross-sectional	58 / 0 / 20	DM: 53.31 ± 12.09, Controls: NR	Serum	ELISA	WHO criteria for T2DM	F2-Isoprostanes: DM ↑ Control	–
Li et al. (2018) [47]	China	Cross-sectional	22 / 0 / 20	DM: 81.76 ± 9.42, Controls: 82.31 ± 10.27	Plasma	ELISA	Clinically diagnosed T2DM	F2-Isoprostanes: DM ↑ Control	–
Nakhjavani et al. (2014) [48]	Iran	Cross-sectional	45 / 0 / 45	DM: 55.18 ± 9.6, Controls: 52.7 ± 11.7	Serum	ELISA	Clinically diagnosed T2DM	F2-Isoprostanes: DM ↑ Control	–
Longo-Mbenza et al. (2014) [41]	DR Congo	Case-control	150 / 0 / 50	DM: 55.2 ± 13, Controls: 50.7 ± 13	Plasma	ELISA	ADA criteria for T2DM	F2-Isoprostanes: DM ↑ Control	–
Wu et al. (2014) [49]	China	Cross-sectional	462 / 0 / 160	DM: 52.59 ± 6.46, Controls: 51.81 ± 6.49	Serum	ELISA	WHO criteria for T2DM	F2-Isoprostanes: DM ↑ Control	–
Tabak et al. (2011) [42]	Turkey	Cross-sectional	69 / 0 / 19	DM: 57.41 ± 8.93, Controls: 52.60 ± 6.64	Serum	ELISA	WHO criteria for T2DM	F2-Isoprostanes: DM ↑ Control	–
Chao et al. (2010) [50]	China	Cross-sectional	118 / 0 / 74	DM: 56.2 ± 9.9, Controls: 52.3 ± 11.7	Plasma	HPLC	Clinically diagnosed T2DM	F2-Isoprostanes: DM ↑ Control	–
Calabrese et al. (2007) [51]	Italy	Cross-sectional	31 / 0 / 16	DM: 60, Controls: 59	Plasma	HPLC	Not stated T2DM	F2-Isoprostanes: DM ↑ Control	–
Feillet-Coudray et al. (2002) [52]	France	Cross-sectional	10 / 0 / 10	DM: 59 ± 7, Controls: 59 ± 6	Plasma	EIA	Not stated T2DM	F2-Isoprostanes: DM − Control	–
Gopaul et al. (1995) [53]	UK	Case-control	39 / 0 / 15	DM: 58.9 ± 2.3, Controls: 33.4 ± 3.1	Plasma	GC-MS	Not stated T2DM	F2-Isoprostanes: DM ↑ Control	–
Loffredo et al. (2020) [54]	Italy	Cross-sectional	0 / 8 / 64	PD: 70 ± 5, Controls: 72 ± 8	Serum	ELISA	NR	–	F2-Isoprostanes: PD ↑ Control
Seet et al. (2010) [55]	Singapore	Case-control	0 / 61 / 61	PD: 64 ± 11.2, Controls: 62.7 ± 5.2	Plasma	GC-MS	UK PD Society Criteria	–	F2-Isoprostanes: PD ↑ Control
Lee et al. (2009) [56]	Singapore	Case-control	0 / 25 / 47	PD: 55.3 ± 7.4, Controls: Age-matched	Plasma	GC-MS	UK PD Society Criteria	–	F2-Isoprostanes: PD − Control
Connolly et al. (2008) [30]	USA	Case-control	0 / 36 / 30	PD: 69 ± 8.7, Controls: Age-matched	Plasma	GC-MS	UK PD Society Criteria	–	F2-Isoprostanes: PD − Control
Irizarry et al. (2007) [57]	USA	Cross-sectional	0 / 47 / 48	PD: 68.2 ± 9.1, Controls: 71.4 ± 9.4	Plasma	GC-MS	NR	–	F2-Isoprostanes: PD − Control

ADA: American Diabetes Association; DM: Diabetes Mellitus; DR Congo: Democratic Republic of the Congo; EIA: Enzyme Immunoassay; ELISA: Enzyme-Linked Immunosorbent Assay; GC-MS: Gas Chromatography-Mass Spectrometry; HPLC: High-Performance Liquid Chromatography; KSA: Kingdom of Saudi Arabia‌; NR: Not Reported; PD: Parkinson’s Disease; T2DM: Type 2 Diabetes Mellitus; UK: United Kingdom; USA: United States of America; WHO: World Health Organization; ↑: Indicates higher levels in the first group compared to the control group; −: Indicates no significant difference between the groups.

**Table 3.** Subgroup analyses for F2-isoprostanes and 8-OHdG in T2DM and PD (mixed effects model).

Disease	Biomarker	Subgroup category	Subgroup	Number of studies	Effect size (Hedges’ g [95% CI])	Heterogeneity (I²)	*P* value
T2DM	F2-Isoprostanes	Age group	<60 years	9	1.69 [1.05, 2.34]	95.8%	<0.001
			≥70 years	2	0.97 [−1.22, 3.15]	96.9%	0.385
		Sample source	Plasma	6	2.38 [1.15, 3.62]	98.3%	<0.001
			Serum	5	0.98 [0.48, 1.48]	88.7%	<0.001
		Assay type	ELISA	8	1.05 [0.67, 1.42]	98.3%	<0.001
			GC-MS	2	4.95 [−5.08, 14.97]	88.7%	0.333
			HPLC	1*	2.98 [2.56, 3.40]	97.2%	<0.001
		Study design	Case-control	2	5.536 [−3.326, 14.398]	98.7%	0.221
			Cohort	1*	−0.116 [−0.305, 0.073]	0.0%	0.230
			Cross-sectional	8	1.303 [0.730, 1.877]	94.2%	<0.001
	8-OHdG	Age group	<60 years	18	3.01 [2.28, 3.74]	97.4%	<0.001
			60–69 years	9	2.01 [1.38, 2.64]	95.1%	<0.001
		DM complications	Diabetic Nephropathy	2	5.24 [2.51, 7.98]	92.8%	<0.001
			Diabetic Retinopathy	2	3.50 [3.13, 3.87]	0.0%	<0.001
		Sample source	Leukocyte DNA	1*	1.54 [1.19, 1.88]	0.0%	<0.001
			Plasma	2	0.47 [0.04, 0.91]	0.0%	0.034
			Serum	24	2.88 [2.32, 3.43]	97.2%	<0.001
		Assay type	EIA (DNA/RNA)	1*	0.80 [0.22, 1.38]	0.0%	0.007
			ELISA	25	2.53 [2.03, 3.04]	96.8%	<0.001
			HPLC-ECD	1*	7.69 [6.37, 9.01]	0.0%	<0.001
		Study design	Case-control	6	2.089 [1.239, 2.939]	94.6%	<0.001
			Cohort	1*	2.752 [2.272, 3.231]	0.0%	<0.001
			Cross-sectional	19	2.942 [2.290, 3.594]	97.5%	<0.001
			Interventional	1*	0.430 [−0.416, 1.275]	0.0%	0.319
PD	F2-Isoprostanes	Study design	Case-control	2	0.784 [−0.868, 2.436]	96.4%	0.352
			Cross-sectional	2	0.061 [−0.372, 0.493]	23.2%	0.783
		Age group	60–69	3	0.498 [−0.647, 1.643]	95.3%	0.394
			≥70	1*	0.408 [−0.330, 1.146]	0.0%	0.279
		Sample source	Plasma	3	0.498 [−0.647, 1.643]	95.3%	0.394
			Serum	1*	0.408 [−0.330, 1.146]	0.0%	0.279
		Assay type	GC-MS	3	0.498 [−0.647, 1.643]	95.3%	0.394
			ELISA	1*	0.408 [−0.330, 1.146]	0.0%	0.279
	8-OHdG	Study design	Case-control	2	0.819 [−0.756, 2.394]	90.3%	0.308
			Cross-sectional	2	0.650 [−0.352, 1.652]	83.0%	0.204
			RCT	1*	1.138 [0.481, 1.794]	0.0%	0.001
		Age group	60–69^a^	5	0.784 [0.180, 1.389]	79.9%	0.011
		Sample source	Serum	4	0.605 [−0.004, 1.215]	78.8%	0.052
			Plasma	1*	1.669 [0.797, 2.541]	0.0%	<0.001
		Assay type	ELISA	3	0.760 [−0.007, 1.528]	83.4%	0.052
			HPLC-ED	2	0.866 [−0.647, 2.379]	87.4%	0.262

8-OHdG: 8-Hydroxy-2’-deoxyguanosine; CI: Confidence Interval; DM: Diabetes Mellitus; DNA: Deoxyribonucleic Acid; EIA: Enzyme Immunoassay; ELISA: Enzyme-Linked Immunosorbent Assay; GC-MS: Gas Chromatography-Mass Spectrometry; HPLC: High-Performance Liquid Chromatography; HPLC-ECD: High-Performance Liquid Chromatography with Electrochemical Detection; PD: Parkinson’s Disease; RCT: Randomized Controlled Trial; RNA: Ribonucleic Acid.

* Indicates subgroups with only one study.

^a^ Indicates that all studies involved participants in the 60–69 age group.

Importantly, PD and T2DM were treated as distinct disease cohorts throughout study selection, data extraction, and meta-analysis, with each condition analyzed separately against healthy control groups rather than as a combined or comorbid population.

### 2.3. Study selection

Two independent reviewers (SSM and MCM) screened titles and abstracts to assess eligibility. Full-text articles were retrieved for studies deemed potentially eligible for inclusion. Discrepancies were resolved through discussion or consultation with a third reviewer. Inclusion focused on human studies reporting blood-based measurements of F2-isoprostanes and 8-OHdG in patients with PD and/or T2DM compared with healthy controls.

### 2.4. Data extraction and synthesis

Two reviewers independently performed data extraction, and discrepancies were resolved through discussion or third-party adjudication. For each eligible study, the following variables were systematically extracted: author(s) and publication year, geographic location, study design, sample size (stratified by T2DM, PD, and control groups), mean age (± standard deviation [SD]), sample source (e.g. hospital or community-based cohorts), assay methodology, diagnostic criteria, and comparative outcomes (T2DM/PD vs. controls). Biochemical measurements were recorded as means and SDs, with exclusive use of baseline (pre-intervention) data to minimize confounding from therapeutic effects. For studies reporting non-parametric data (medians with interquartile ranges [IQRs] or ranges), validated conversion formulas were applied to approximate means and SDs: mean ≈ median + (lower bound + upper bound − 2 × median)/4 and SD ≈ (upper bound - lower bound)/4, as recommended by Hozo et al. [36] and Wan et al. [37] for meta-analytic standardization. For studies with multiple subgroups (e.g. diabetic patients with and without complications), subgroup means were combined using weighted averages, and pooled SDs were calculated according to standard meta-analytic procedures [38].

### 2.5. Critical appraisal of included studies

The methodological quality of the included studies was assessed using the appropriate Joanna Briggs Institute (JBI) critical appraisal tools for each study design: the checklist for analytical cross-sectional studies (*n* = 34), the checklist for case-control studies (*n* = 16), the checklist for cohort studies (*n* = 2), and the checklist for quasi-experimental studies (*n* = 1) [39]. For the randomized controlled trial (RCT) (*n* = 1), the risk of bias was assessed using the risk of bias 2 (ROB 2) tool [40]. The appraisal focused on reporting quality, external validity, and internal validity, including the assessment of bias and confounding. Quality assessments were conducted independently by SSM and MCM, with disagreements resolved through discussion.

### 2.6. Statistical analysis

All measurements were standardized to nanograms per milliliter (ng/mL) before analysis. Meta-analyses were conducted using standardized mean differences (Hedges’ g) and their 95% confidence intervals. Heterogeneity was assessed using the DerSimonian-Laird estimator. We performed comprehensive sensitivity analyses to evaluate the robustness of the pooled effect size estimates for F2-isoprostanes and 8-OHdG. First, we conducted a leave-one-out sensitivity analysis within the primary random-effects model to assess the influence of individual studies on the summary estimates by iteratively removing one study at a time and recalculating the pooled effect size. Second, we applied Duval and Tweedie’s trim-and-fill method within a random-effects framework to adjust for potential publication bias by imputing theoretically missing studies and estimating adjusted pooled effect sizes. Heterogeneity was quantified using I^2^ (the percentage of variation due to heterogeneity) and τ^2^ (the between-study variance) for each model, except for the trim-and-fill method, where heterogeneity statistics reflect those of the original random-effects model. All statistical analyses were done using Comprehensive Meta-Analysis (CMA) version 3.0 software and SPSS version 29.

## 3. Results

### 3.1. Search results

**Figure 1.**
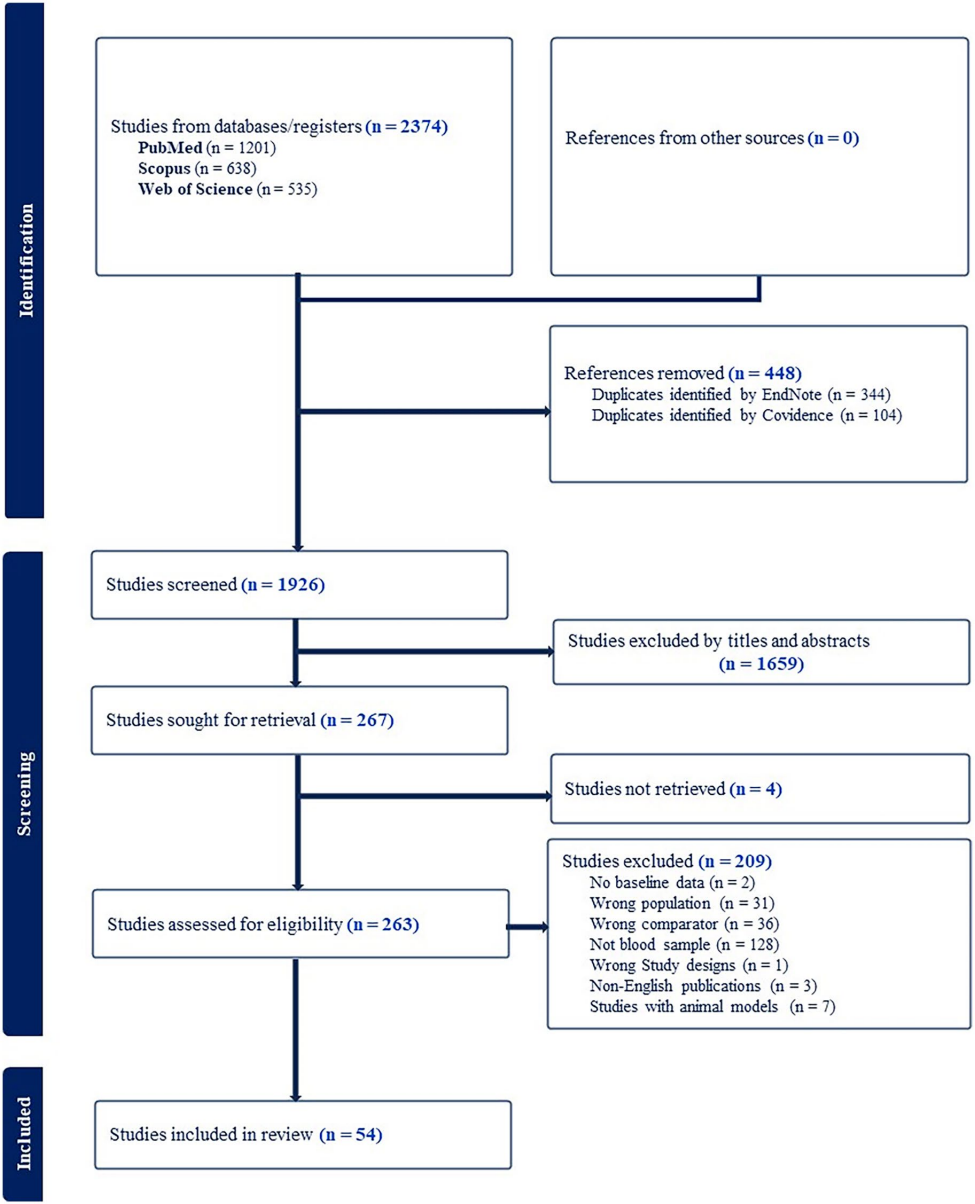
PRISMA flow diagram illustrating the selection process of studies for this review.

The initial database search yielded 2374 records, with 1201 identified in PubMed, 535 in Web of Science, and 638 in Scopus. Following deduplication – using EndNote (344 duplicates removed) and Covidence (an additional 104 duplicates eliminated) – the remaining unique records were subjected to title and abstract screening. Full-text articles were retrieved for studies meeting the eligibility criteria. The detailed search strategy is provided in the supplementary materials, and a PRISMA flow diagram summarizing the study selection process is included in Figure 1.

All subsequent analyses were conducted as disease-specific comparisons of PD and T2DM cohorts relative to their respective control groups; no direct or comorbid comparisons between PD and T2DM populations were performed.

### 3.2. Study characteristics

This systematic review identified 18 studies examining F2-isoprostanes (13 in T2DM, 5 in PD; total *n* = 2807 participants [T2DM 1262, PD 177, Control 1368]) and 38 studies assessing 8-OHdG (30 in T2DM, 8 in PD; total *n* = 4719 participants [T2DM 2260, PD 545, Control 1914]). Two studies assessed both F2-isoprostanes and 8-OHdG, contributing to both analyses [41,42].

These investigations spanned diverse geographical regions, including North America, Europe, Asia, Africa, and the Middle East (Tables 1 and 2), with a quality index ranging from moderate to high (Supplementary Tables S4–S8).

**Table 2.** Summary of studies on 8-OHdG in Diabetes Mellitus and Parkinson’s disease.

Author (Year)	Location	Study design	Sample size (DM/PD/Controls)	Mean age (Years)	Biomarkers	Sample source	Assay type (8-OHdG Only)	Diagnostic criteria	Results (Summary of DM vs Control on 8-OHdG)	Results (Summary of PD vs Control on 8-OHdG)
Tajane et al. (2024) [58]	India	Case-control	50 / 0 / 50	DM: 53.08 ± 10.4, Controls: 42.98 ± 9.61	8-OHdG	Serum	ELISA	WHO and ADA (2018) T2DM	8-OHdG: DM ↑ Control	–
Lazutka et al. (2024) [59]	Lithuania	Interventional pre-post	8 / 0 / 16	DM: 53.2 ± 15.4, Controls: 44.6 ± 10.5	8-OHdG	Plasma	ELISA	Not specified T2DM	8-OHdG: DM ↑ Control	–
Corbacho Alonso et al. (2023) [60]	Spain	Cross-sectional	18 / 0 / 20	DM: 72 ± 9, Controls: 71 ± 9	8-OHdG	Plasma	ELISA	Not specified T2DM	8-OHdG: DM − Control	–
Nemtsova et al. (2022) [61]	Ukraine	Cohort	156 / 0 / 30	DM: 62.11 ± 0.64, Controls: age-matched	8-OHdG	Serum	ELISA	Not specified T2DM	8-OHdG: DM ↑ Control	–
Wang et al. (2022) [62]	China	Cross-sectional	94 / 0 / 100	DM: 65.8 ± 7.0, Controls: 64.4 ± 8.1	8-OHdG	Serum	ELISA	Ophthalmology (3rd edition) criteria T2DM	8-OHdG: DM ↑ Control	–
Attia et al. (2021) [63]	Iraq	Cross-sectional	45 / 0 / 45	DM: 54.44 ± 11.19, Controls: 49.21 ± 14.91	8-OHdG	Serum	ELISA	Diagnosed clinically with T2DM	8-OHdG: DM ↑ Control	–
Mahmoud et al. (2021) [64]	Iraq	Cross-sectional	297 / 0 / 188	DM: 52.9 ± 8.5, Controls: 47.0 ± 8.7	8-OHdG	Serum	ELISA	ADA Criteria T2DM	8-OHdG: DM ↑ Control	–
Abudawood et al. (2020) [65]	KSA	Cross-sectional	100 / 0 / 50	DM: 56.86 ± 14.52, Control: 49.77 ± 16.67	8-OHdG	Serum	ELISA	ADA Criteria T2DM	8-OHdG: DM ↑ Control	–
Goycheva et al. (2019) [66]	Bulgaria	Cross-sectional	93 / 0 / 94	DM: 64.2 ± 9.8, Controls: 56.6 ± 11.2	8-OHdG	Serum	ELISA	WHO criteria for T2DM	8-OHdG: DM ↑ Control	–
Nemtsova et al. (2018) [67]	Ukraine	Cross-sectional	126 / 0 / 20	DM: 57.8 ± 6.2, Controls: age-matched	8-OHdG	Serum	ELISA	Ukrainian Ministry of Health orders T2DM	8-OHdG: DM ↑ Control	–
Silva et al. (2018) [68]	Brazil	Cross-sectional	27 / 0 / 133	DM: 68.3 ± 5.60 Controls: age-matched	8-OHdG	Serum	ELISA	Diagnosed clinically with T2DM	8-OHdG: DM ↑ Control	–
Alaaraj et al. (2018) [69]	Iraq	Cross-sectional	51 / 0 / 31	DM: 51.00 ± 6.90, Controls: 49.00 ± 8.90	8-OHdG	Serum	ELISA	Diagnosed clinically T2DM	8-OHdG: DM ↑ Control	–
Mansour et al. (2018) [70]	Egypt	Cross-sectional	150 / 0 / 50	DM: 58.1 ± 4.4, Controls: 56 ± 3	8-OHdG	Serum	ELISA	Diagnosed clinically with T2DM	8-OHdG: DM ↑ Control	–
El Horany et al. (2017) [71]	Egypt	Cross-sectional	45 / 0 / 15	DM: 55.5 ± 3.8, Controls: 54.9 ± 5.1	8-OHdG	Serum	ELISA	ADA criteria for T2DM	8-OHdG: DM ↑ Control	–
Ye et al. (2016) [72]	China	Case-control	108 / 0 / 65	DM: 60 ± 17, Controls: 55 ± 11	8-OHdG	Leukocyte	ELISA	WHO criteria T2DM	8-OHdG: DM ↑ Control	–
Nasif et al. (2016) [73]	KSA and Egypt	Cross-sectional	17 / 0 / 50	DM: 50 ± 8, Controls: 27 ± 4	8-OHdG	Serum	ELISA	ADA criteria for T2DM	8-OHdG: DM ↑ Control	–
Sun et al. (2015) [74]	China	Case-control	28 / 0 / 65	DM: 50 ± 8, Controls: 27 ± 4	8-OHdG	Serum	ELISA	WHO criteria T2DM	8-OHdG: DM ↑ Control	–
Hasan & Mohieldein (2015) [75]	KSA	Case-control	60 / 0 / 60	DM: 56.54 ± 9.5, Controls: 45.66 ± 11.3	8-OHdG	Serum	ELISA	Diagnosed clinically with T2DM	8-OHdG: DM ↑ Control	–
Ravassa et al. (2015) [76]	Spain	Case-control	72 / 0 / 14	DM: 65.3 ± 8.6, Controls: 63.4 ± 8.7	8-OHdG	Serum	EIA	ADA criteria for T2DM	8-OHdG: DM ↑ Control	–
Longo-Mbenza et al. (2014) [41]	DR Congo	Case-control	150 / 0 / 50	DM: 55.2 ± 13, Controls: 50.7 ± 13	8-OHdG	Serum	ELISA	ADA criteria for T2DM	8-OHdG: DM ↑ Control	–
Mahfouz et al. (2012) [77]	Egypt	Cross-sectional	60 / 0 / 20	DM: 52.6 ± 1.44, Controls: 49.7 ± 1.87	8-OHdG	Serum	ELISA	ADA criteria for T2DM	8-OHdG: DM ↑ Control	–
Letonja et al. (2012) [78]	Slovenia	Cross-sectional	75 / 0 / 70	DM: 62.75 ± 9.77, Controls: 59.78 ± 9.16	8-OHdG	Serum	ELISA	WHO criteria for T2DM	8-OHdG: DM ↑ Control	–
Al Aubaidy & Jelinek (2011) [79]	Australia	Cross-sectional	35 / 0 / 119	DM: 69.9 ± 8.6, Controls: 66.4 ± 11.1	8-OHdG	Serum	ELISA	Diagnosed clinically with T2DM	8-OHdG: DM ↑ Control	–
Tabak et al. (2011) [42]	Turkey	Cross-sectional	69 / 0 / 19	DM: 57.41 ± 8.93, Controls: 52.60 ± 6.64	8-OHdG	Plasma	ELISA	WHO criteria for T2DM	8-OHdG: DM − Control	–
Chen et al. (2011) [80]	China	Case-control	66 / 0 / NR	DM: 61.0 ± 16.3, Controls: NR	8-OHdG	Peripheral blood leukocytes	ELISA	WHO criteria for T2DM	8-OHdG: DM ↑ Control	–
Al Aubaidy & Jelinek (2010) [81]	Australia	Cross-sectional	35 / 0 / 98	DM: 70 ± 8.4, Controls: 66.2 ± 11.2	8-OHdG	Serum	ELISA	ADA criteria for T2DM	8-OHdG: DM ↑ Control	–
Pan et al. (2010) [82]	China	Case-control	77 / 0 / 40	DM: 51.80 ± 11.87, Controls: 52.38 ± 11.12	8-OHdG	Serum	ELISA	ADA criteria for T2DM	8-OHdG: DM ↑ Control	–
Pan et al. (2008) [83]	China	Cross-sectional	60 / 0 / 32	DM: 51.36 ± 12.66, Controls: 53.22 ± 11.64	8-OHdG	Serum	ELISA	WHO criteria for T2DM	8-OHdG: DM ↑ Control	–
Pan et al. (2007) [84]	China	Cross-sectional	47 / 0 / 25	DM: 58, Controls: 58	8-OHdG	Serum	ELISA	WHO criteria for T2DM	8-OHdG: DM ↑ Control	–
Shin et al. (2001) [85]	Korea	Cross-sectional	41 / 0 / 33	DM: 57.2 ± 2.1, Controls: 51.7 ± 1.4	8-OHdG	Serum	HPLC-ECD	Diagnosed clinically with T2DM	8-OHdG: DM ↑ Control	–
Tsai et al. (2025) [86]	China	RCT	0 / 41 / 20	PD: 64.85 ± 7.46, Controls: 67.00 ± 7.07	8-OHdG	Serum	ELISA	Diagnosis confirmed by a movement disorders specialist	–	8-OHdG: PD ↑ Control
Gmitterová et al. (2018) [87]	Germany	Case-control	0 / 44 / 32	PD: 65.30 ± 7.71, Controls: 65.25 ± 8.75	8-OHdG	Serum	ELISA	MDS criteria for PD	–	8-OHdG: PD – Control
Bolner et al. (2011) [88]	Italy	Case-control	0 / 13 / 13	All: 68 ± 12	8-OHdG	Plasma	HPLC-ED	NR	–	8-OHdG: PD ↑ Control
Chen et al. (2009) [24]	China	Case-control	0 / 211 / 135	PD: 67.3 ± 0.7, Controls: 68.4 ± 1.0	8-OHdG	Leukocyte	HPLC-ED	GOG criteria for PD	–	8-OHdG: PD ↑ Control
Bogdanov et al. (2008) [89]	USA	Case-control	0 / 66 / 25	PD: 66.0 ± 11.1, Controls: 61.5 ± 12.2	8-OHdG	Plasma	HPLC-ED	Based on Pankratz et al. (2002)	–	8-OHdG: PD ↑ Control
Dorszewska et al. (2007) [90]	Poland	Cross-sectional	0 / 98 / 50	PD: 60.8 ± 10.7, Controls: 44.6 ± 16.2	8-OHdG	Leukocyte	HPLC/ED/UV	UK PD Society Criteria	–	8-OHdG: PD – Control
Abe et al. (2003) [91]	Japan	Cross-sectional	0 / 24 / 15	PD: 63.3 ± 10.5, Controls: 62.3 ± 9.4	8-OHdG	Serum	HPLC-ED	Koller’s criteria for PD	–	8-OHdG: PD – Control
Kikuchi et al. (2002) [92]	Japan	Cross-sectional	0 / 48 / 22	PD: 65.75 ± 9.74, Controls: 60.54 ± 8.76	8-OHdG	Serum	ELISA	Koller’s criteria for PD	–	8-OHdG: PD ↑ Control

ADA: American Diabetes Association; DM: Diabetes Mellitus; DR Congo: Democratic Republic of the Congo; EIA: Enzyme Immunoassay; ELISA: Enzyme-Linked Immunosorbent Assay; GOG: Gelb, Oliver, and Gilman; HPLC/ED/UV: High-Performance Liquid Chromatography with Electrochemical Detection and Ultraviolet Detection; HPLC-ECD: High-Performance Liquid Chromatography with Electrochemical Detection; HPLC-ED: High-Performance Liquid Chromatography with Electrochemical Detection; KSA: Kingdom of Saudi Arabia; MDS: Movement Disorder Society; NR: Not Reported; PD: Parkinson’s Disease; RCT: Randomized Controlled Trial; T2DM: Type 2 Diabetes Mellitus; UK: United Kingdom; USA: United States of America; WHO: World Health Organization; ↑: Indicates higher levels in the first group (DM or PD) compared to the control group; −: Indicates no significant difference between the groups.

#### 3.2.1. F2-isoprostanes

- Study designs and sample sizes: Among T2DM studies, cross-sectional designs predominated (10/13), with sample sizes ranging from 10 to 462 participants (median: 66). PD studies, all employing case-control or cross-sectional designs, were smaller, with sample sizes ranging from 8 to 61 participants (median: 36).
- Sample sources and assay types: In T2DM studies, plasma (8/13) and serum (5/13) were the primary sample matrices. Assay methods included enzyme-linked immunosorbent assay (ELISA; 8/13), gas chromatography-mass spectrometry (GC-MS; 2/13), high-performance liquid chromatography (HPLC; 2/13), and enzyme immunoassay (EIA; 1/13). For PD studies, plasma was the predominant sample source (4/5), with assays conducted via GC–MS (3/5) and ELISA (2/5).
- Diagnostic criteria: Diagnostic criteria in T2DM studies included World Health Organization (WHO) guidelines (4/13), American Diabetes Association (ADA) standards (1/13), clinical diagnoses (5/13), or unspecified protocols (3/13). PD studies utilized the United Kingdom PD Society Brain Bank criteria (3/5), with two studies not specifying diagnostic standards.

#### 3.2.2. 8-OhdG

- Study designs and sample sizes: T2DM studies were predominantly cross-sectional (20/30), with sample sizes ranging from 8 to 297 participants (median: 60). PD studies, employing various designs including case-control and cross-sectional approaches, ranged from 13 to 211 participants (median: 44).
- Sample sources and assay types: Serum (25/30) and plasma (3/30) were the primary sample sources in T2DM studies, with ELISA as the dominant assay method (28/30). In PD studies, sample sources included serum (4/8), plasma (2/8), and leukocyte DNA (2/8), with assays performed using ELISA (3/8) or HPLC-based methods (5/8).
- Diagnostic criteria: T2DM studies applied WHO criteria (19/30), ADA criteria (8/30), clinical diagnoses (8/30), or unspecified criteria. PD studies utilized movement disorder specialist diagnoses (1/8), Movement Disorder Society criteria (1/8), or other diagnostic protocols.

Demographic characteristics varied across studies. T2DM cohorts exhibited a mean age range of 50–81.8 years. PD participants were generally older (mean age: 55.3–70 years), though sex-specific data were inconsistently provided. Methodological heterogeneity arose from differences in assay techniques (e.g. ELISA, GC-MS, HPLC-ECD, and sample matrices (e.g. serum, plasma, leukocytes), contributing to substantial between-study variability. These demographic differences were considered within disease-specific analyses rather than as inter-disease comparisons.

### 3.3. Systematic review and meta-analysis of F2-isoprostanes

The systematic review identified 18 studies on F2-isoprostanes, comprising 13 in T2DM and 5 in PD. Among the T2DM studies, 11 reported significantly elevated F2-isoprostane levels compared to controls, while two found no significant difference (Table 1). In PD cohorts, evaluated independently, findings were heterogeneous; two of five studies identified elevated levels, whereas three observed no significant difference. The type of assay appeared to influence these outcomes. In T2DM, studies using GC–MS yielded variable results (e.g. one study reported no difference, while another detected an elevation). These methodological patterns were assessed within each disease group rather than as direct inter-disease contrasts.

**Figure 2.**
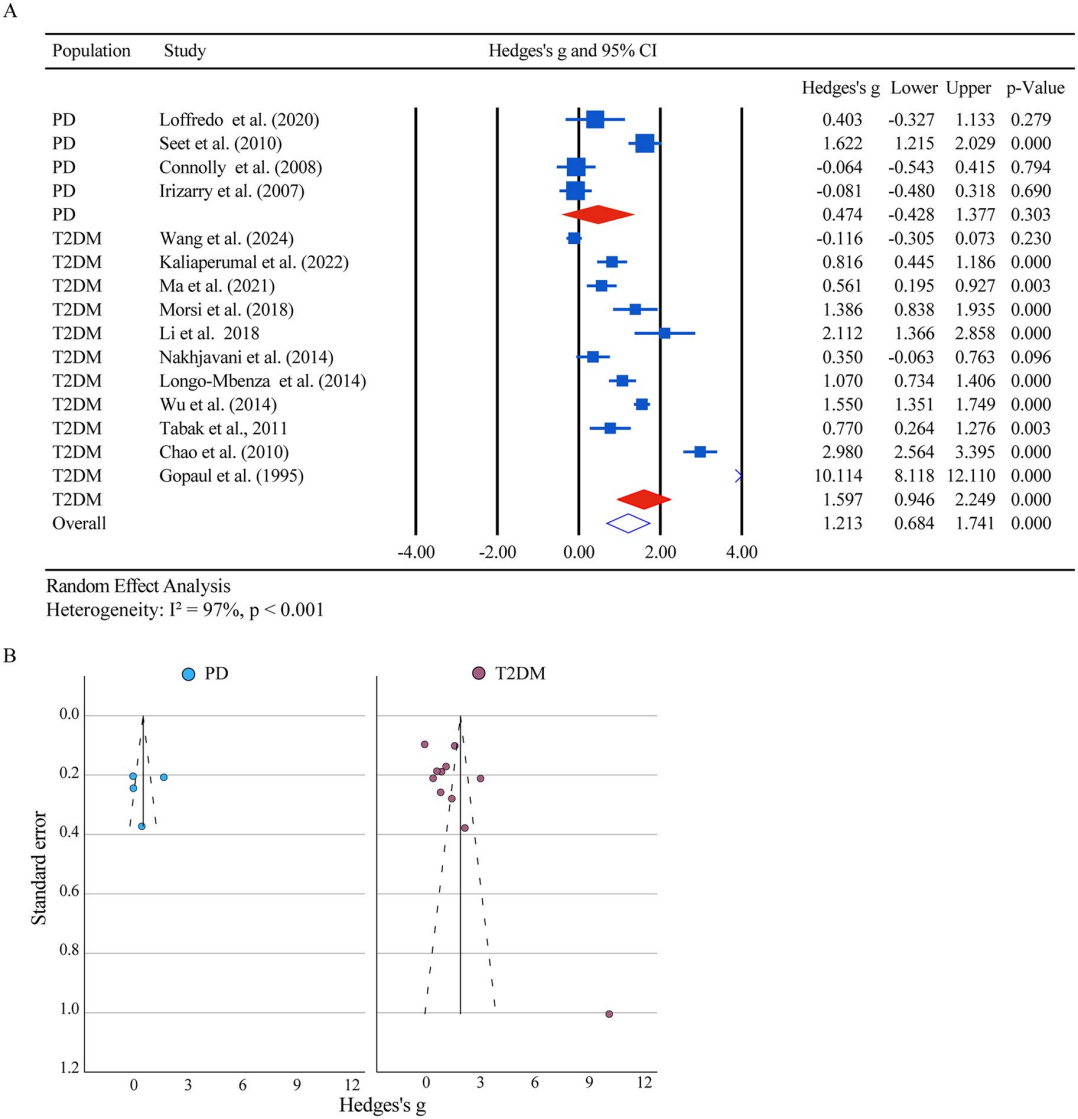
Meta‑analysis and publication bias assessment of F2‑Isoprostane levels in T2DM and PD. (A) Forest plot showing pooled effect sizes and 95 % confidence intervals for F2‑isoprostane levels; comparisons between T2DM *vs.* controls, PD *vs.* controls, and the overall combined estimate. (B) Funnel plot displaying study effect sizes against standard errors for F2‑isoprostane levels in T2DM and PD; each point represents one study.

To quantify these observations, a meta-analysis was conducted on 15 of the 18 studies that provided sufficient data for analysis. The analysis revealed a significant overall increase in F2-isoprostane levels (Hedges’ *g* = 1.21, 95% CI: 0.68–1.74; *p* < 0.001), though this was accompanied by extreme heterogeneity (I^2^ ≈ 97%) (Figure 2A). Subgroup analysis demonstrated a significant effect in T2DM (*g* = 1.60, 95% CI: 0.95–2.25; *p* < 0.001; 11 studies), whereas no statistically significant pooled effect was observed within PD cohorts (*g* = 0.47, 95% CI: −0.43 to 1.38; *p* = 0.303; 4 studies). In PD, further subgroup analyses by study design, age group, sample source, and assay type did not yield significant results, with high heterogeneity observed in some subgroups (e.g. I^2^ = 95.3% for the 60–69 age group) (Table 3). Specifically, analyses by study design showed no significant differences for case-control (*g* = 0.78, 95% CI: −0.87 to 2.44; *p* = 0.35; 2 studies) or cross-sectional studies (*g* = 0.06, 95% CI: −0.37 to 0.49; *p* = 0.78; 2 studies). Similarly, stratification by age group indicated no significant effects for the 60–69 age group (*g* = 0.50, 95% CI: −0.65 to 1.64; *p* = 0.39; 3 studies) or the >70 age group (*g* = 0.41, 95% CI: −0.33 to 1.15; *p* = 0.28; 1 study). Subgroup analyses by sample source and assay type were also non-significant (Table 3). In T2DM, further stratification by sample source indicated more significant effects in plasma (*g* = 2.38, 95% CI: 1.15–3.62) compared to serum and in cohorts with a mean age under 60 years (*g* = 1.69, 95% CI: 1.05–2.34) compared to older cohorts (Table 3).

### 3.4. Systematic review and meta-analysis of 8-OHdG

The systematic review identified 38 studies on 8-OHdG, comprising 30 in T2DM and 8 in PD. In T2DM, 28 of 30 studies demonstrated significantly elevated 8-OHdG levels compared to controls, with only two reporting no significant difference (Table 2). In PD cohorts, analyzed separately, five of eight studies demonstrated significant elevations, generally of smaller magnitude. Serum-based assays predominated in T2DM studies (25 of 30), and HPLC-based methods tended to yield larger effect sizes than ELISA.

**Figure 3.**
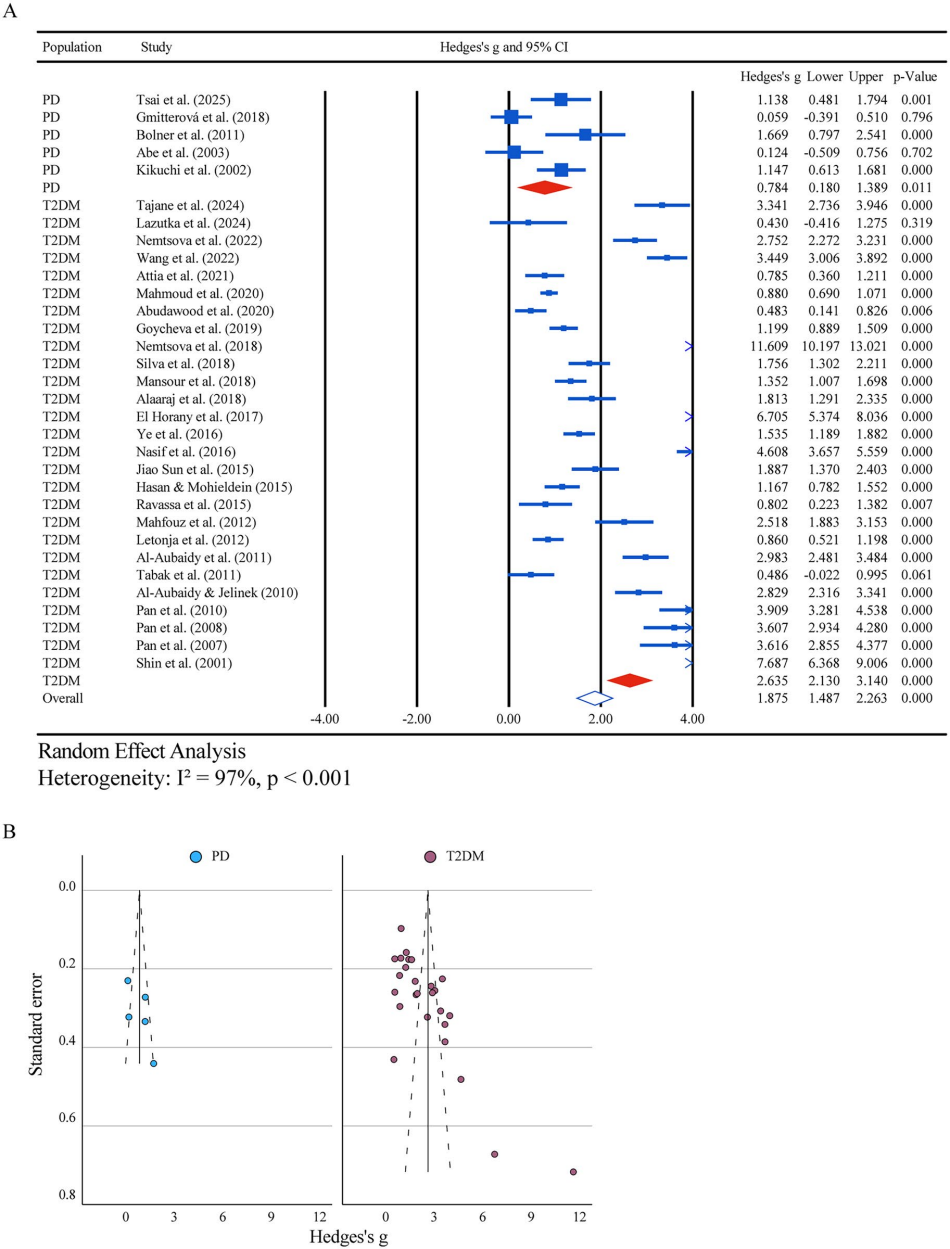
Meta‑analysis and publication bias assessment of 8‑OHdG levels in T2DM and PD. (A) Forest plot showing pooled effect sizes and 95 % confidence intervals for 8‑OHdG levels; T2DM *vs.* controls, PD *vs.* controls, and the overall combined estimate. (B) Funnel plot showing study effect sizes against standard errors for 8‑OHdG in T2DM and PD; each bullet denotes a distinct study.

Building on these findings, a meta-analysis was performed on 32 of the 38 studies that provided sufficient data. The pooled analysis revealed a significant increase in 8-OHdG levels (*g* = 1.88, 95% CI: 1.49–2.26; *p* < 0.001), with pronounced heterogeneity (I^2^ ≈ 97%) (Figure 3A). Subgroup analysis showed a strong effect in T2DM (*g* = 2.64, 95% CI: 2.13–3.14; *p* < 0.001; 27 studies) and a moderate increase in PD (*g* = 0.78, 95% CI: 0.18–1.39; *p* = 0.011; 5 studies). In PD, subgroup analyses by study design indicated a significant effect in randomized controlled trials (RCTs) (*g* = 1.14, 95% CI: 0.48 to 1.79; *p* = 0.001; 1 study) but not in case-control (*g* = 0.82, 95% CI: −0.76 to 2.39; *p* = 0.31; 2 studies) or cross-sectional studies (*g* = 0.65, 95% CI: −0.35 to 1.65; *p* = 0.20; 2 studies). All PD studies fell within the 60–69 age group, exhibiting a significant overall effect (*g* = 0.784, 95% CI: 0.180–1.389; *p* = 0.011; 5 studies). Further analyses by sample source revealed a significant elevation in plasma (*g* = 1.67, 95% CI: 0.80 to 2.54; *p* < 0.001; 1 study) but not in serum (*g* = 0.61, 95% CI: −0.004 to 1.215; *p* = 0.05; 4 studies). Subgroup analyses by assay type showed no significant effects for ELISA (*g* = 0.76, 95% CI: −0.01 to 1.53; *p* = 0.05; 3 studies) or HPLC-ED (*g* = 0.87, 95% CI: −0.65 to 2.38; *p* = 0.26; 2 studies) (Table 3). In T2DM, subgroup analyses identified more significant effects in serum samples (*g* = 2.88, 95% CI: 2.32–3.43) compared to other matrices and in cohorts with a mean age under 60 years (*g* = 3.01, 95% CI: 2.28–3.74) relative to older groups (Table 3). Additionally, studies focusing on diabetic complications reported substantial effect sizes for Diabetic Nephropathy (DN; *g* = 5.24, 95% CI: 2.51–7.98; *p* < 0.001; 2 studies) and Diabetic Retinopathy (DR; *g* = 3.50, 95% CI: 3.13–3.87; *p* < 0.001; 2 studies), though DN exhibited high heterogeneity (I^2^ = 92.8%) while DR showed none (I^2^ = 0.0%) (Table 3).

### 3.5. Risk of bias and sensitivity analysis

Egger’s regression tests revealed significant small-study effects in T2DM cohorts, indicating potential publication bias (F2-isoprostanes: SEb = 10.10, *p* < 0.001; 8-OHdG: SEb = 13.15, *p* < 0.001), whereas no bias was detected in PD subgroups (Figures 2B and 3B). Sensitivity analyses confirmed the stability of pooled effect size estimates for both biomarkers (Supplementary Table S9). For F2-isoprostanes, the leave-one-out sensitivity analysis, conducted within a random-effects framework, indicated a stable pooled effect size with a representative estimate of 1.277 (95% CI: 0.746–1.809; I^2^ = 96.55%), while the fixed-effects model showed a lower estimate of 0.847 (95 giving a single estimate (95% CI: 0.754–0.940). The trim-and-fill method, adjusting for one imputed study, produced an effect size of 0.941 (95% CI: 0.371–1.510). For 8-OHdG, the leave-one-out sensitivity analysis within a random-effects framework resulted in a representative pooled effect size of 2.367 (95% CI: 1.908–2.825; I^2^ = 96.59%), compared to 1.547 (95% CI: 1.465–1.629) under fixed-effects assumptions. The trim-and-fill adjustment, imputing eight studies, yielded an effect size of 1.364 (95% CI: 0.845–1.883). High I^2^ values reflect substantial between-study heterogeneity, and trim-and-fill adjustments suggest modest publication bias. All bias and sensitivity assessments were conducted separately for PD and T2DM cohorts, reinforcing the robustness of disease-specific findings while supporting cautious interpretation.

## 4. Discussion

This systematic review and meta-analysis compiled evidence on the oxidative stress biomarkers F2-isoprostanes and 8-OHdG in T2DM and PD. Our findings demonstrate significant and consistent increases in both biomarkers in individuals with T2DM, with large effect sizes (Hedges’*g* ≈ 1.60 for F2-isoprostanes and *g* ≈ 2.64 for 8-OHdG) – in contrast to PD, where a moderate increase in 8-OHdG (*g* ≈ 0.78) was observed, and F2-isoprostanes exhibited only a modest, non-significant change (*g* ≈ 0.47). These divergent oxidative profiles highlight the differential contributions of lipid peroxidation and DNA damage under these conditions, providing a comparative framework to explore their potential comorbidity. Given that most included studies were observational, these findings should be interpreted as associations rather than evidence of causality; mechanistic interpretations are therefore inferential and hypothesis-generating.

### 4.1. Oxidative stress in T2DM

The significant increase in F2-isoprostanes and 8-OHdG levels in T2DM is consistent with prior evidence linking chronic hyperglycemia to elevated oxidative stress [18,93]. In T2DM, hyperglycemia-induced oxidative stress has been associated with lipid peroxidation and DNA oxidation, which are thought to contribute to the development of macrovascular and microvascular complications [18]. Subgroup analyses further indicate that T2DM patients with complications, such as diabetic nephropathy and retinopathy, tend to exhibit even more significant effect sizes, supporting an association between disease progression and oxidative burden. These findings align with prior meta-analyses that report elevated oxidative stress markers in metabolic disorders, supporting the use of these biomarkers for risk stratification and monitoring of T2DM, although their clinical application remains to be prospectively validated [94,95].

### 4.2. Oxidative stress in PD

In contrast to the pronounced systemic oxidative stress observed in T2DM, the pattern in PD appears more variable and compartmentalized. The moderate elevation in 8-OHdG is consistent with oxidative DNA damage being associated with dopaminergic neurodegeneration [29,96,97]. However, this relationship should be interpreted as associative rather than causal. The absence of a significant increase in F2-isoprostanes suggests that lipid peroxidation may be less prominent or more variable in PD. This variability could reflect the localized nature of lipid peroxidation in the central nervous system (CNS) or differences in disease stage among patients with PD and potentially other neurodegenerative diseases, warranting further investigation [7,98,99]. This discrepancy highlights the compartmentalized nature of oxidative stress in neurodegeneration, suggesting that blood-based biomarkers may not fully capture oxidative processes within the CNS [100,101].

Subgroup analyses of F2-isoprostanes in PD revealed no significant differences across study designs, age groups, sample sources, or assay types, highlighting the intricate nature of oxidative stress in this condition. For 8-OHdG, significant effects were observed in RCT and plasma samples but not in other study designs or sample sources. This suggests that methodological factors may influence biomarker detection and that these findings should be interpreted cautiously. This discrepancy highlights the compartmentalized nature of oxidative processes in neurodegeneration, suggesting that peripheral blood-based biomarkers may not accurately reflect CNS-specific oxidative damage [100,101]. Consequently, while oxidative stress is implicated in PD, its expression appears more heterogeneous than the systemic oxidative burden seen in T2DM. These distinct oxidative profiles may inform hypotheses regarding the interplay between T2DM and PD, although direct evidence from comorbid populations is currently lacking.

### 4.3. Implications for PD-T2DM comorbidity

The differing oxidative profiles in T2DM and PD raise hypotheses about potential mechanistic overlaps between these conditions. Epidemiological studies have consistently suggested that T2DM increases the risk of developing PD and may accelerate its progression [12–14,20]. Shared pathogenic features – including mitochondrial dysfunction, insulin resistance, and neuroinflammation – may be linked to elevated ROS levels, although direct mechanistic connections in comorbid patients remain to be established. This pro-oxidative environment has been associated with lipid peroxidation of cellular membranes, as indicated by increased levels of F2-isoprostanes, and induces oxidative damage to DNA, as reflected by elevated 8-OHdG levels. Taken together, these parallel findings suggest a potential overlap in oxidative pathways; however, this inference is indirect and remains to be validated in explicitly comorbid populations [9–11,102].

The consistent elevation of 8-OHdG in both T2DM and PD, particularly its significant increase in PD RCTs and plasma samples, supports the hypothesis that oxidative DNA damage may represent a convergent feature of these conditions, warranting further investigation in explicitly comorbid populations [103,104]. Additionally, the divergent findings for F2-isoprostanes across PD subgroups underscore the need to investigate whether these biomarkers can serve as early indicators of PD-T2DM comorbidity, although this remains speculative. If validated in longitudinal studies, early alterations in these markers could potentially inform risk assessment or therapeutic timing, but prospective clinical studies are required. Emerging evidence suggests that antidiabetic agents add clinical relevance to these hypotheses, yet direct applications remain to be established [20]. Future studies should focus on comorbid populations to determine whether systemic oxidative stress in T2DM exacerbates PD-related neurodegeneration, or vice versa, thereby guiding the development of targeted therapeutic approaches.

### 4.4. Methodological considerations and future directions

The high heterogeneity (I^2^ ≈ 97%) observed across studies reflects variability in assay methods (e.g. ELISA, GC-MS, HPLC-ECD), sample sources (serum vs. plasma), and patient demographics. These methodological inconsistencies complicate direct comparisons of oxidative stress measurements, highlighting the pressing need for standardized protocols and suggesting that caution is warranted when interpreting pooled effect sizes. The lack of significant differences in F2-isoprostanes across PD subgroups (e.g. study design, age, sample source, assay type) and the selective significance of 8-OHdG in RCTs and plasma samples further illustrate how methodological factors may obscure biomarker detection.

Future research should employ multi-compartment sampling, including cerebrospinal fluid, to capture both systemic and CNS-specific oxidative stress, thereby addressing whether peripheral markers accurately reflect brain oxidative damage. A multi-modal approach integrating biochemical, neuroimaging, clinical, and electrophysiological data is also essential for a holistic understanding of PD-T2DM comorbidity. Such an approach could confirm whether early alterations in F2-isoprostanes and 8-OHdG predict subsequent neurodegenerative processes. Refining assay techniques – balancing cost-effective methods like ELISA with precise methodologies like GC-MS – is crucial for elucidating the subtle dynamics of oxidative damage in PD, particularly in the context of T2DM comorbidity. Longitudinal studies are further required to clarify the temporal patterns of redox dysregulation and validate these biomarkers as early predictors of PD-T2DM comorbidity.

### 4.5. Limitations

Despite the strengths of this meta-analysis, several limitations must be acknowledged. First, the predominance of cross-sectional studies precludes causal inferences, meaning that observed associations should not be interpreted as evidence of mechanistic links. The underrepresentation of PD studies – especially those reporting F2 isoprostanes – limits the statistical power for detecting subgroup differences. Second, no data are available to characterize how F2-isoprostanes and 8-OHdG vary across different disease stages, genders, or clinical phenotypes of PD. This prevents any assessment of biomarker trajectories over disease progression. Third, variability in diagnostic criteria and assay methodologies introduces additional confounding, and potential publication bias in T2DM studies may inflate effect sizes. Nonetheless, the consistency of T2DM findings and the absence of significant bias in PD data strengthen the reliability of our conclusions.

An additional limitation is the restriction of included studies to participants aged ≥50 years. Aging is independently associated with increased oxidative stress and may have amplified biomarker levels irrespective of disease status. Consequently, these findings may not generalize to younger populations with T2DM or PD, in whom oxidative stress dynamics could differ. Future studies should examine age-stratified cohorts to clarify whether biomarker patterns are disease-specific across the lifespan.

Finally, while this review highlights potential clinical and translational relevance of F2-isoprostanes and 8-OHdG, these applications remain speculative and hypothesis-generating until validated in prospective or comorbid cohorts.

## 5. Conclusion

This comparative analysis demonstrates that oxidative stress biomarkers – particularly 8-OHdG – are consistently elevated in T2DM, while PD exhibits a more modest oxidative signature. Given that the studies included are predominantly observational, these findings reflect associations rather than mechanistic causation. The potential of F2-isoprostanes and 8-OHdG as early indicators of PD-T2DM comorbidity remains hypothetical and requires further investigation in explicitly comorbid populations. Any overlap in oxidative pathways, including features related to mitochondrial dysfunction and insulin resistance, should be interpreted as an indirect observation rather than a confirmed causal link. Future studies should prioritize comorbid populations with standardized assays, multi-compartment sampling, and multi-modal longitudinal designs.

Additionally, research should consider age-stratified cohorts as the current evidence is largely restricted to participants aged ≥50 years, which may limit generalizability to younger populations. Such approaches will be essential to clarify whether systemic oxidative stress is associated with bidirectional pathology and to evaluate the prospective utility of these biomarkers in guiding targeted, personalized interventions.

## Supplementary Material

Supplementary material.docx

PRISMA_2020_checklist.docx

## Data Availability

The PRISMA checklist for this article is deposited in Figshare (Title: PRISMA_2020_checklist for Comparative Evaluation of Oxidative Stress Biomarkers F2-Isoprostanes and 8-OHdG in Parkinson’s Disease and Type 2 Diabetes Mellitus: A Systematic Review and Meta-Analysis of Human Studies; Doi:10.6084/m9.figshare.30356386; License: CC0) [105]. The data that support the findings of this study are included in the article/Supplementary material. Further inquiries can be directed to the corresponding authors.

## Open scholarship

This article has earned the Center for Open Science badges for Open Data, Open Materials and Preregistered+. The data and materials are openly accessible at https://doi.org/10.6084/m9.figshare.30356386, https://doi.org/10.6084/m9.figshare.30356386 and https://www.crd.york.ac.uk/PROSPERO/view/CRD42025633253
