# Supplementary material for: Comparative evaluation of oxidative stress biomarkers F2-isoprostanes and 8-OHdG in Parkinson’s disease and Type 2 Diabetes Mellitus: a systematic review and meta-analysis of human studies
Source: Ann Med. 2026 Apr 20;58(1):2654251. doi: 10.1080/07853890.2026.2654251 (PMC13097182; doi:10.1080/07853890.2026.2654251)
Supplement: Supplementary material.docx [file IANN_A_2654251_SM2180.docx]

**Table. S1: Full search strategy in PUBMED (December 20, 2024)**

| Line | | Keywords | Records  retrieved |
| --- | --- | --- | --- |
| #1  Participants | Parkinson’s Disease OR Diabetes Mellitus | "Parkinson Disease"[MeSH Terms] OR "parkinson*"[Title/Abstract] OR "PD"[Title/Abstract] OR "Diabetes Mellitus"[MeSH Terms] OR "Diabetes"[Title/Abstract] OR "DM"[Title/Abstract] | 1,199,769 |
| #2  Intervention (and respective outcomes) | Blood-based F2-IsoPs and/or 8-OHdG levels. | "Mitochondrial Dysfunction"[Title/Abstract] OR "Oxidative Stress"[Mesh] OR "F2-isoprostane"[Title/Abstract] OR "8 isoprostane"[Title/Abstract] OR "8-iso-PGF2alpha"[Title/Abstract] OR "8-epi-PGF2alpha"[Title/Abstract] OR "15-F2t-isoprostane"[Title/Abstract] OR "iPF2alpha-III"[Title/Abstract] OR "8-hydroxy-2'-deoxyguanosine"[Title/Abstract] OR "8-OHdG"[Title/Abstract] | 216,029 |
| #3  Comparator | Healthy control subjects | "Control"[Title/Abstract] OR "Healthy Control"[Title/Abstract] | 3,344,786 |
| #4 | ((#1) AND (#2)) AND (#3) | ("Parkinson Disease"[MeSH Terms] OR "parkinson*"[Title/Abstract] OR "PD"[Title/Abstract] OR ("Diabetes Mellitus"[MeSH Terms] OR "Diabetes"[Title/Abstract] OR "DM"[Title/Abstract])) AND ("Mitochondrial Dysfunction"[Title/Abstract] OR "Oxidative Stress"[MeSH Terms] OR "F2-isoprostane"[Title/Abstract] OR "8 isoprostane"[Title/Abstract] OR "8-iso-PGF2alpha"[Title/Abstract] OR "8-epi-PGF2alpha"[Title/Abstract] OR "15-F2t-isoprostane"[Title/Abstract] OR "iPF2alpha-III"[Title/Abstract] OR "8 hydroxy 2 deoxyguanosine"[Title/Abstract] OR "8-OHdG"[Title/Abstract]) AND ("Control"[Title/Abstract] OR "Healthy Control"[Title/Abstract]) | 6,036 |
| #5 | Animal studies | (animals [mh] NOT humans [mh]) | 5,289,610 |
| #6  NOT Animal studies | (#4) NOT (#5) | (("Parkinson Disease"[MeSH Terms] OR "parkinson*"[Title/Abstract] OR "PD"[Title/Abstract] OR ("Diabetes Mellitus"[MeSH Terms] OR "Diabetes"[Title/Abstract] OR "DM"[Title/Abstract])) AND ("Mitochondrial Dysfunction"[Title/Abstract] OR "Oxidative Stress"[MeSH Terms] OR "F2-isoprostane"[Title/Abstract] OR "8 isoprostane"[Title/Abstract] OR "8-iso-PGF2alpha"[Title/Abstract] OR "8-epi-PGF2alpha"[Title/Abstract] OR "15-F2t-isoprostane"[Title/Abstract] OR "iPF2alpha-III"[Title/Abstract] OR "8 hydroxy 2 deoxyguanosine"[Title/Abstract] OR "8-OHdG"[Title/Abstract]) AND ("Control"[Title/Abstract] OR "Healthy Control"[Title/Abstract])) NOT ("animals"[MeSH Terms] NOT "humans"[MeSH Terms]) | 3,020 |
| #7 | in vitro studies | "in vitro" OR cellular OR CRISPR OR immunoblotting OR knockout OR cell* OR culture | 10,377,176 |
| #8  NOT in vitro studies | (#6) NOT (#7) | ((("Parkinson Disease"[MeSH Terms] OR "parkinson*"[Title/Abstract] OR "PD"[Title/Abstract] OR ("Diabetes Mellitus"[MeSH Terms] OR "Diabetes"[Title/Abstract] OR "DM"[Title/Abstract])) AND ("Mitochondrial Dysfunction"[Title/Abstract] OR "Oxidative Stress"[MeSH Terms] OR "F2-isoprostane"[Title/Abstract] OR "8 isoprostane"[Title/Abstract] OR "8-iso-PGF2alpha"[Title/Abstract] OR "8-epi-PGF2alpha"[Title/Abstract] OR "15-F2t-isoprostane"[Title/Abstract] OR "iPF2alpha-III"[Title/Abstract] OR "8 hydroxy 2 deoxyguanosine"[Title/Abstract] OR "8-OHdG"[Title/Abstract]) AND ("Control"[Title/Abstract] OR "Healthy Control"[Title/Abstract])) NOT ("animals"[MeSH Terms] NOT "humans"[MeSH Terms])) NOT ("in vitro"[All Fields] OR ("cells"[MeSH Terms] OR "cells"[All Fields] OR "cellular"[All Fields]) OR ("clustered regularly interspaced short palindromic repeats"[MeSH Terms] OR ("clustered"[All Fields] AND "regularly"[All Fields] AND "interspaced"[All Fields] AND "short"[All Fields] AND "palindromic"[All Fields] AND "repeats"[All Fields]) OR "clustered regularly interspaced short palindromic repeats"[All Fields] OR "crispr"[All Fields] OR "crisprs"[All Fields]) OR ("immunobloting"[All Fields] OR "immunoblots"[All Fields] OR "immunoblotted"[All Fields] OR "immunoblotting"[MeSH Terms] OR "immunoblotting"[All Fields] OR "immunoblot"[All Fields] OR "immunoblottings"[All Fields]) OR ("knockout"[All Fields] OR "knockouts"[All Fields]) OR "cell*"[All Fields] OR ("culturabilities"[All Fields] OR "culturability"[All Fields] OR "culturable"[All Fields] OR "culturalism"[All Fields] OR "culture"[MeSH Terms] OR "culture"[All Fields] OR "cultural"[All Fields] OR "culturally"[All Fields] OR "cultures"[All Fields] OR "culture s"[All Fields] OR "cultured"[All Fields] OR "culturing"[All Fields] OR "culturings"[All Fields] OR "ethnology"[MeSH Subheading] OR "ethnology"[All Fields])) | 1,201 |

**Table. S2: Full search strategy in Web of Science (December 20, 2024)**

| Line | | Keywords | Records  retrieved |
| --- | --- | --- | --- |
| #1  Participants | Parkinson’s Disease OR Diabetes Mellitus | TS= ("Parkinson Disease" OR "Parkinson*" OR "Diabetes Mellitus" OR "Diabetes" OR "Type 2 Diabetes" OR "Type 1 Diabetes") | 1,480,891 |
| #2  Intervention (and respective outcomes) | Blood-based F2-IsoPs / 8-OHdG levels. | TS= ("F2-isoprostane" OR "8-isoprostane" OR "8-iso-PGF2α" OR "8-epi-PGF2α" OR "15-F2t-isoprostane" OR "iPF2α-III" OR "8-hydroxy-2'-deoxyguanosine" OR "8-OHdG") | 16,027 |
| #3  Comparator | Healthy control subjects | TS= ("Control" OR "Healthy Control") | 9,254,106 |
| #4 | #1 AND #2 AND #3 | #1 AND #2 AND #3 | 6,036 |
| #5 | Animal studies | TS= (((animal* OR rat OR rats OR mouse OR mice OR murine OR dog OR dogs OR canine OR cat OR cats OR feline OR rabbit OR cow OR cows OR bovine OR rodent* OR sheep OR ovine OR pig OR swine OR porcine OR veterinar* OR chick* OR zebrafish* OR baboon* OR nonhuman* OR primate* OR cattle* OR goose OR geese OR duck OR macaque* OR avian* OR bird* OR fish*) NOT (human* OR patient* OR women OR woman OR men OR man))) | 6,895,305 |
| #6  NOT Animal studies | (#4) NOT (#5) | (#4) NOT #5 | 535 |

**Table. S3: Full search strategy in Scopus (December 20, 2024)**

| Line | | Keywords | Records  retrieved |
| --- | --- | --- | --- |
| #1  Participants | Parkinson’s Disease OR Diabetes Mellitus | TITLE-ABS-KEY ("Diabetes Mellitus" OR "Diabetes" OR "Type 2 Diabetes" OR "Type 1 Diabetes" OR "Parkinson Disease" OR "Parkinson*") | 1,478,840 |
| #2  Intervention (and respective outcomes) | Blood-based F2-IsoPs and/or 8-OHdG levels. | TITLE-ABS-KEY ("F2-isoprostane" OR "8-isoprostane" OR "8-iso-PGF2α" OR "8-epi-PGF2α" OR "15-F2t-isoprostane" OR "iPF2α-III" OR "8-hydroxy-2-deoxyguanosine" OR "8-OHdG") | 12,503 |
| #3  Comparator | Healthy control subjects | TITLE-ABS-KEY ("Control" OR "Healthy Control") | 10,506,604 |
| #4 | 1 AND 2 AND 3 | (TITLE-ABS-KEY ("Diabetes Mellitus" OR "Diabetes" OR "Type 2 Diabetes" OR "Type 1 Diabetes" OR "Parkinson Disease" OR "Parkinson*")) AND (TITLE-ABS-KEY ("F2-isoprostane" OR "8-isoprostane" OR "8-iso-PGF2α" OR "8-epi-PGF2α" OR "15-F2t-isoprostane" OR "iPF2α-III" OR "8-hydroxy-2-deoxyguanosine" OR "8-OHdG")) AND (TITLE-ABS-KEY ("Control" OR "Healthy Control")) | 638 |

**Table. S4: Summary of JBI critical appraisal for the included cross-sectional studies[1]**

| Study ID | Author (Year) | Inclusion criteria defined | Study subjects and setting described | Exposure measured reliably | Standard criteria for condition measurement | Confounders identified | Strategies to address confounders | Outcomes measured reliably | Statistical analysis appropriate | Overall appraisal | Comments |
| --- | --- | --- | --- | --- | --- | --- | --- | --- | --- | --- | --- |
| 1 | Corbacho Alonso et al. (2023)[2] | Yes | Yes | Yes | Yes | Yes | Yes | Yes | Yes | Include | High quality |
| 2 | Wang et al. (2022)[3] | Yes | Yes | Yes | Yes | Yes | Yes | Yes | Yes | Include | High quality |
| 3 | Kaliaperumal et al. (2022)[4] | Yes | Yes | Yes | Yes | Yes | Yes | Yes | Yes | Include | High quality |
| 4 | Ma et al. (2021)[5] | Yes | Yes | Yes | Yes | Yes | Yes | Yes | Yes | Include | High quality |
| 5 | Attia et al. (2021)[6] | Yes | Yes | Yes | Yes | Yes | No | Yes | Yes | Include | Moderate quality |
| 6 | Loffredo et al. (2020)[7] | Yes | Yes | Yes | Unclear | Yes | Yes | Yes | Yes | Include | High quality |
| 7 | Mahmoud et al. (2021)[8] | Yes | Yes | Yes | Yes | Yes | Yes | Yes | Yes | Include | High quality |
| 8 | Abudawood et al. (2020)[9] | Yes | Yes | Yes | Yes | Yes | Yes | Yes | Yes | Include | High quality |
| 9 | Goycheva et al. (2019)[10] | Yes | Yes | Yes | Yes | Yes | No | Yes | Yes | Include | Moderate quality |
| 10 | Morsi et al. (2018)[11] | Yes | Yes | Yes | Yes | Unclear | Unclear | Yes | Yes | Include | Moderate quality |
| 11 | Li et al. (2018)[12] | Yes | Yes | Yes | Yes | Yes | Yes | Yes | Yes | Include | High quality |
| 12 | Nemtsova et al. (2018)[13] | Yes | Yes | Yes | Yes | Yes | Yes | Yes | Yes | Include | High quality |
| 13 | Silva et al. (2018)[14] | Yes | Yes | Yes | Yes | Yes | Yes | Yes | Yes | Include | High quality |
| 14 | Alaaraj et al. (2018)[15] | Yes | Yes | Yes | Yes | Yes | No | Yes | Yes | Include | Moderate quality |
| 15 | Mansour et al. (2018)[16] | Yes | Yes | Yes | Yes | Unclear | Unclear | Yes | Yes | Include | Moderate quality |
| 16 | El Horany et al. (2017)[17] | Yes | Yes | Yes | Yes | Yes | Yes | Yes | Yes | Include | High quality |
| 17 | Nasif et al. (2016)[18] | Yes | Yes | Yes | Yes | Yes | Unclear | Yes | Yes | Include | Moderate quality |
| 18 | Nakhjavani et al. (2014)[19] | Yes | Yes | Yes | Yes | Yes | Yes | Yes | Yes | Include | High quality |
| 19 | Wu et al. (2014)[20] | Yes | Yes | Yes | Yes | Yes | Yes | Yes | Yes | Include | High quality |
| 20 | Mahfouz et al. (2012)[21] | Yes | Yes | Yes | Yes | Yes | Unclear | Yes | Yes | Include | Moderate quality |
| 21 | Letonja et al. (2012)[22] | Yes | Yes | Yes | Yes | Yes | Yes | Yes | Yes | Include | High quality |
| 22 | Tabak et al. (2011)[23] | Yes | Yes | Yes | Yes | Yes | Yes | Yes | Yes | Include | High quality |
| 23 | Al Aubaidy et al. (2011)[24] | Yes | Yes | Yes | Yes | Yes | Yes | Yes | Yes | Include | High quality |
| 24 | Chao et al. (2010)[25] | Yes | Yes | Yes | Yes | Yes | Yes | Yes | Yes | Include | High quality |
| 25 | Pan et al. (2008)[26] | Yes | Yes | Yes | Yes | Yes | Unclear | Yes | Yes | Include | Moderate quality |
| 26 | Al Aubaidy et al. (2010)[27] | Yes | Yes | Yes | Yes | Yes | Yes | Yes | Yes | Include | High quality |
| 27 | Calabrese et al. (2007)[28] | Yes | Yes | Yes | Yes | Yes | Yes | Yes | Yes | Include | High quality |
| 28 | Irizarry et al. (2007)[29] | Yes | Yes | Yes | Yes | Yes | Yes | Yes | Yes | Include | High quality |
| 29 | Dorszewska et al. (2007)[30] | Yes | Yes | Yes | Yes | Unclear | Unclear | Yes | Yes | Include | Moderate quality |
| 30 | Pan et al. (2007)[31] | Yes | Yes | Yes | Yes | Yes | Yes | Yes | Yes | Include | High quality |
| 31 | Abe et al. (2003)[32] | Yes | Yes | Yes | Yes | Unclear | Unclear | Yes | Yes | Include | Moderate quality |
| 32 | Feillet-Coudray et al. (2002)[33] | Yes | Yes | Yes | Yes | Yes | Unclear | Yes | Yes | Include | Moderate quality |
| 33 | Kikuchi et al. (2002)[34] | Yes | Yes | Yes | Yes | Yes | Unclear | Yes | Yes | Include | Moderate quality |
| 34 | Shin et al. (2001)[35] | Yes | Yes | Yes | Yes | Yes | No | Yes | Yes | Include | Moderate quality |

**Table. S5: Summary of JBI critical appraisal for the included case-control studies[1]**

| Study ID | Author (Year) | Groups comparable | Cases & controls matched | Same criteria for identification | Exposure measured reliably | Exposure measured same way for both groups | Confounders identified | Strategies to address confounders | Outcomes assessed reliably | Exposure period meaningful | Statistical analysis appropriate | Overall appraisal | Comments |
| --- | --- | --- | --- | --- | --- | --- | --- | --- | --- | --- | --- | --- | --- |
| 1 | Tajane et al. (2024)[36] | No | No | Yes | Yes | Yes | Yes | Yes | Yes | Yes | Yes | Include | Moderate quality |
| 2 | Gmitterová et al. (2018)[37] | Yes | Unclear | Unclear | Yes | Yes | Yes | Yes | Yes | Yes | Yes | Include | Moderate Quality |
| 3 | Ye et al. (2016)[38] | Yes | Yes | Yes | Yes | Yes | Yes | Yes | Yes | Yes | Yes | Include | Moderate quality |
| 4 | Sun et al. (2015)[39] | Unclear | No | Yes | Yes | Yes | Unclear | No | Yes | Yes | Yes | Include | Moderate quality |
| 5 | Hasanet al. (2015)[40] | Yes | Yes | Yes | Yes | Yes | Yes | No | Yes | Yes | Yes | Include | Moderate quality |
| 6 | Ravassa et al. (2015)[41] | Yes | Yes | Yes | Yes | Yes | Yes | Yes | Yes | Yes | Yes | Include | High quality |
| 7 | Longo-Mbenza et al. (2014)[42] | Yes | Yes | Yes | Yes | Yes | Yes | Unclear | Yes | Yes | Yes | Include | Moderate quality |
| 8 | Bolner et al. (2011)[43] | Yes | Yes | Yes | Yes | Yes | Yes | Yes | Yes | Yes | Yes | Include | Moderate-to-high quality |
| 9 | Chen et al. (2011)[44] | Yes | Yes | Yes | Yes | Yes | Yes | Yes | Yes | Yes | Yes | Include | High Quality |
| 10 | Pan et al. (2010)[45] | Yes | Yes | Yes | Yes | Yes | Yes | Yes | Yes | Yes | Yes | Include | Moderate quality |
| 11 | Seet et al. (2010)[46] | Yes | Yes | Yes | Yes | Yes | Yes | Yes | Yes | Yes | Yes | Include | High quality |
| 12 | Chen et al. (2009)[47] | Yes | No | Yes | Yes | Yes | Yes | Yes | Yes | Yes | Yes | Include | High quality |
| 13 | Lee et al. (2009)[48] | Yes | Yes | Yes | Yes | Yes | Yes | Yes | Yes | Yes | Yes | Include | High quality |
| 14 | Bogdanov et al. (2008)[49] | Yes | Yes | Yes | Yes | Yes | Yes | Yes | Yes | Yes | Yes | Include | High quality |
| 15 | Connolly et al. (2008)[50] | Yes | Yes | Yes | Yes | Yes | Yes | Yes | Yes | Yes | Yes | Include | High quality |
| 16 | Gopaul et al. (1995)[51] | Yes | Yes | Yes | Yes | Yes | Yes | Unclear | Yes | Yes | Yes | Include | Moderate quality |

**Table. S6: Summary of JBI critical appraisal for the included cohort studies[1]**

| Study ID | Author | Year | Record number | Q1 | Q2 | Q3 | Q4 | Q5 | Q6 | Q7 | Q8 | Q9 | Q10 | Q11 | Overall appraisal | Comments |
| --- | --- | --- | --- | --- | --- | --- | --- | --- | --- | --- | --- | --- | --- | --- | --- | --- |
| 1 | Wang et al.[52] | 2024 | 1 | Yes | Yes | Yes | Yes | Yes | Unclear | Yes | Yes | Unclear | Unclear | Yes | Include | High quality |
| 2 | Nemtsova et al.[13] | 2022 | 2 | Yes | Yes | Yes | Yes | Yes | Yes | Yes | Yes | Yes | Yes | Yes | Include | High quality |

Q1: Were the two groups similar and recruited from the same population?

Q2: Were the exposures measured similarly to assign people to both exposed and unexposed groups?

Q3: Was the exposure measured validly and reliably?

Q4: Were confounding factors identified?

Q5: Were strategies to deal with confounding factors stated?

Q6: Were the groups/participants free of the outcome at the start of the study?

Q7: Were the outcomes measured validly and reliably?

Q8: Was the follow-up time reported and sufficient for outcomes to occur?

Q9: Was follow-up complete, and if not, were reasons for loss to follow-up described and explored?

Q10: Were strategies to address incomplete follow-up utilized?

Q11: Was appropriate statistical analysis used?

**Table. S7: Summary of JBI critical appraisal for the included quasi-experimental (interventional pre-post) study[1]**

| Study | Clear causal relationship? | Participants similar? | Control group used? | Multiple measurements? | Outcome measures reliable? | Appropriate statistical analysis? | Overall quality |
| --- | --- | --- | --- | --- | --- | --- | --- |
| Lazutka et al. (2024)[53] | Yes | Partial | Yes | Yes | Yes | Yes | Moderate quality |

**Table. S8: Summary of risk of bias (ROB 2) assessment for the included RCT[54]**

| Study ID | Domain 1: Randomization | Domain 2: Deviations from intended interventions | Domain 3: Missing data | Domain 4: Outcome measurement | Domain 5: Reporting | Overall ROB Judgment | Comments on overall quality |
| --- | --- | --- | --- | --- | --- | --- | --- |
| Tsai et al. (2025)[55] | Low Risk | Low Risk | Low Risk | Low Risk | Low Risk | Low Risk | High quality |

Table. S9: Risk of bias and sensitivity analysis

| **Sensitivity Analysis** | **F2-isoprostanes Pooled ES (95% CI)** | **F2-isoprostanes I² (%)** | **F2-isoprostanes τ²** | **8-OHdG Pooled ES (95% CI)** | **8-OHdG I² (%)** | **8-OHdG τ²** |
| --- | --- | --- | --- | --- | --- | --- |
| Leave-one-out analysis | 1.277 (0.746, 1.809) | 96.55 | 1.012 | 2.367 (1.908, 2.825) | 96.59 | 1.639 |
| Fixed effects model | 0.847 (0.754, 0.940) | 96.55 | 0 | 1.547 (1.465, 1.629) | 96.59 | 0 |
| Trim and fill (random effects) | 0.941 (0.371, 1.510) * | — | — | 1.364 (0.845, 1.883) * | — | — |

*Adjusted for publication bias (1 study imputed for F2-isoprostanes; 8 studies imputed for 8-OHdG). Q-values (F2-isoprostanes: 489.235; 8-OHdG: 1612.342 for adjusted).

**References**

1. Aromataris, E. and Z. Munn, *JBI manual for evidence synthesis*. 2020: Jbi.

2. Corbacho-Alonso, N., et al., *Global Oxidative Status Is Linked to Calcific Aortic Stenosis: The Differences Due to Diabetes Mellitus and the Effects of Metformin.* Antioxidants (Basel), 2023. **12**(5): p. 1024.

3. Wang, X.L., et al., *Changes and significance of retinal blood oxygen saturation and oxidative stress indexes in patients with diabetic retinopathy.* World J Diabetes, 2022. **13**(5): p. 408-416.

4. Kaliaperumal, R., et al., *Association of Serum 8-isoprostaglandin F2α Levels with Glycemic Control in Type 2 Diabetes Patients with Senile Cataract.* Journal of the Indian Medical Association, 2022. **120**(10): p. 34-38.

5. Ma, N., et al., *Associations of plasma 8-iso-prostaglandin F(2alpha)levels with fasting blood glucose (FBG) and intra-abdominal fat (IAF) area in various Glycometabolism populations.* BMC Endocr Disord, 2021. **21**(1): p. 215.

6. Attia, Z.M., H.J. Hammood, and M.A. Assi, *Assessment of Oxidative Stress Biomarker (8-OHdG) and Paraoxonases 1 in Type II Diabetic Mellitus.* International Journal of Drug Delivery Technology, 2021. **11**(4): p. 1389-1393.

7. Loffredo, L., et al., *Oxidative Stress and Gut-Derived Lipopolysaccharides in Neurodegenerative Disease: Role of NOX2.* Oxid Med Cell Longev, 2020. **2020**: p. 8630275.

8. Mahmoud, H.M., A.F. Ali, and D.J. Al-Timimi, *Relationship Between Zinc Status and DNA Oxidative Damage in Patients with Type 2 Diabetes Mellitus.* Biol Trace Elem Res, 2021. **199**(4): p. 1276-1279.

9. Abudawood, M., et al., *Interrelationship between oxidative stress, DNA damage and cancer risk in diabetes (Type 2) in Riyadh, KSA.* Saudi J Biol Sci, 2020. **27**(1): p. 177-183.

10. Goycheva, P., et al., *Predictive value of some pro-oxidants in type 2 diabetes mellitus with vascular complications.* Biosci Trends, 2019. **13**(2): p. 168-175.

11. Morsi, H.K. and M.M. Ismail, *The value of 8-iso prostaglandin F2 alpha and superoxide dismutase activity as a clinical indicator of oxidative stress in type II diabetes mellitus.* Journal of Clinical and Diagnostic Research, 2018. **12**(11): p. BC10-BC14.

12. Li, J. and X. Shen, *Leptin concentration and oxidative stress in diabetic ketoacidosis.* Eur J Clin Invest, 2018. **48**(10): p. e13006.

13. Nemtsova, V., et al., *Role of plasma 8-OXO-2'-deoxyguanosine in target organ damage in patients with hypertension and type 2 diabetes.* Arterial Hypertension (Poland), 2022. **26**(2): p. 78-83.

14. da Silva, L.C., et al., *Evaluation of oxidative DNA damage in elderly patients with type 2 diabetes mellitus living in ivoti, state of Rio grande do Sul.* Acta Scientiarum - Health Sciences, 2018. **40**: p. e37813.

15. Alaaraji, S.F.T., et al., *Evaluation of serum 8-Hydroxy-Deoxyguanosine, interleukin-6 and interleukin-7 in type 2 diabetic iraqi females.* Journal of Global Pharma Technology, 2018. **10**(7): p. 473-480.

16. Mansour, M.M., et al., *Serum 8-hydroxydeoxyguanosine and aldose reductase C-106T polymorphism in type 2 diabetes mellitus and its relation to complications in Egyptian patients.* Comparative Clinical Pathology, 2018. **27**(1): p. 99-105.

17. El-Horany, H.E., et al., *NLRP3 expression and urinary HSP72 in relation to biomarkers of inflammation and oxidative stress in diabetic nephropathy patients.* IUBMB Life, 2017. **69**(8): p. 623-630.

18. Nasif, W.A., et al., *Oxidative DNA damage and oxidized low density lipoprotein in Type II diabetes mellitus among patients with Helicobacter pylori infection.* Diabetol Metab Syndr, 2016. **8**: p. 34.

19. Nakhjavani, M., et al., *Prostaglandin F2 alpha plasma concentration predicts glycemic control and oxidation status in patients with type 2 diabetes mellitus.* Clin Lab, 2014. **60**(12): p. 2073-80.

20. Wu, C., et al., *The changes of serum sKlotho and NGAL levels and their correlation in type 2 diabetes mellitus patients with different stages of urinary albumin.* Diabetes Res Clin Pract, 2014. **106**(2): p. 343-50.

21. Mahfouz, M.H., I.A. Emara, and G.A. Omar, *Biomarkers of oxidative DNA damage and soluble Fas/Fas ligand in type 2 diabetic patients.* American Journal of Applied Sciences, 2012. **9**(4): p. 450-458.

22. Letonja, M.S., et al., *Association of the C242T polymorphism in the NADPH oxidase p22 phox gene with carotid atherosclerosis in Slovenian patients with type 2 diabetes.* Mol Biol Rep, 2012. **39**(12): p. 10121-30.

23. Tabak, O., et al., *Oxidative lipid, protein, and DNA damage as oxidative stress markers in vascular complications of diabetes mellitus.* Clin Invest Med, 2011. **34**(3): p. E163-71.

24. Al-Aubaidy, H.A. and H.F. Jelinek, *Oxidative DNA damage and obesity in type 2 diabetes mellitus.* Eur J Endocrinol, 2011. **164**(6): p. 899-904.

25. Chao, P.C., et al., *Association of dietary AGEs with circulating AGEs, glycated LDL, IL-1alpha and MCP-1 levels in type 2 diabetic patients.* Eur J Nutr, 2010. **49**(7): p. 429-34.

26. Pan, H.Z., et al., *The change of oxidative stress products in diabetes mellitus and diabetic retinopathy.* Br J Ophthalmol, 2008. **92**(4): p. 548-51.

27. Al-Aubaidy, H.A. and H.F. Jelinek, *8-Hydroxy-2-deoxy-guanosine identifies oxidative DNA damage in a rural prediabetes cohort.* Redox Rep, 2010. **15**(4): p. 155-60.

28. Calabrese, V., et al., *Oxidative stress and cellular stress response in diabetic nephropathy.* Cell Stress Chaperones, 2007. **12**(4): p. 299-306.

29. Irizarry, M.C., et al., *Plasma F2A isoprostane levels in Alzheimer's and Parkinson's disease.* Neurodegener Dis, 2007. **4**(6): p. 403-5.

30. Dorszewska, J., et al., *Oxidative DNA damage and level of thiols as related to polymorphisms of MTHFR, MTR, MTHFD1 in Alzheimer's and Parkinson's diseases.* Acta Neurobiol Exp (Wars), 2007. **67**(2): p. 113-29.

31. Pan, H.Z., et al., *Oxidative damage to DNA and its relationship with diabetic complications.* Biomed Environ Sci, 2007. **20**(2): p. 160-3.

32. Abe, T., et al., *Alteration of 8-hydroxyguanosine concentrations in the cerebrospinal fluid and serum from patients with Parkinson's disease.* Neurosci Lett, 2003. **336**(2): p. 105-8.

33. Feillet-Coudray, C., et al., *Divergence in plasmatic and urinary isoprostane levels in type 2 diabetes.* Clin Chim Acta, 2002. **324**(1-2): p. 25-30.

34. Kikuchi, A., et al., *Systemic increase of oxidative nucleic acid damage in Parkinson's disease and multiple system atrophy.* Neurobiol Dis, 2002. **9**(2): p. 244-8.

35. Shin, C.S., et al., *Serum 8-hydroxy-guanine levels are increased in diabetic patients.* Diabetes Care, 2001. **24**(4): p. 733-7.

36. Tajane, T., et al., *Serum oxidative stressors levels and association of mtDNA variants with type 2 diabetes mellitus in the Central India population.* Human Gene, 2024. **42**: p. 201337.

37. Gmitterova, K., et al., *DNA versus RNA oxidation in Parkinson's disease: Which is more important?* Neurosci Lett, 2018. **662**: p. 22-28.

38. Ye, X., et al., *Increased 8-hydroxy-2'-deoxyguanosine in leukocyte DNA from patients with type 2 diabetes and microangiopathy.* J Int Med Res, 2016. **44**(3): p. 472-82.

39. Sun, J., et al., *Serum 8-hydroxy-2′-deoxyguanosine (8-oxo-dG) levels are elevated in diabetes patients.* International Journal of Diabetes in Developing Countries, 2015. **35**(3): p. 368-373.

40. Hasan, M. and A. Mohieldein, *Association between serum carcinoembryonic antigen level and oxidative stress parameters among diabetic females.* Int J Clin Exp Med, 2015. **8**(4): p. 6489-94.

41. Ravassa, S., et al., *Association of low GLP-1 with oxidative stress is related to cardiac disease and outcome in patients with type 2 diabetes mellitus: a pilot study.* Free Radic Biol Med, 2015. **81**: p. 1-12.

42. Longo-Mbenza, B., et al., *Retinopathy in non diabetics, diabetic retinopathy and oxidative stress: a new phenotype in Central Africa?* Int J Ophthalmol, 2014. **7**(2): p. 293-301.

43. Bolner, A., et al., *Plasma and urinary HPLC-ED determination of the ratio of 8-OHdG/2-dG in Parkinson's disease.* Clin Lab, 2011. **57**(11-12): p. 859-66.

44. Chen, H., et al., *AluYb8 insertion in the MUTYH gene is related to increased 8-OHdG in genomic DNA and could be a risk factor for type 2 diabetes in a Chinese population.* Mol Cell Endocrinol, 2011. **332**(1-2): p. 301-5.

45. Pan, H.Z., et al., *The oxidative stress status in diabetes mellitus and diabetic nephropathy.* Acta Diabetol, 2010. **47 Suppl 1**: p. 71-6.

46. Seet, R.C., et al., *Oxidative damage in Parkinson disease: Measurement using accurate biomarkers.* Free Radic Biol Med, 2010. **48**(4): p. 560-6.

47. Chen, C.M., et al., *Increased oxidative damage in peripheral blood correlates with severity of Parkinson's disease.* Neurobiol Dis, 2009. **33**(3): p. 429-35.

48. Lee, C.Y., et al., *Different patterns of oxidized lipid products in plasma and urine of dengue fever, stroke, and Parkinson's disease patients: cautions in the use of biomarkers of oxidative stress.* Antioxid Redox Signal, 2009. **11**(3): p. 407-20.

49. Bogdanov, M., et al., *Metabolomic profiling to develop blood biomarkers for Parkinson's disease.* Brain, 2008. **131**(Pt 2): p. 389-96.

50. Connolly, J., et al., *F2 isoprostane levels in plasma and urine do not support increased lipid peroxidation in cognitively impaired Parkinson disease patients.* Cogn Behav Neurol, 2008. **21**(2): p. 83-6.

51. Gopaul, N.K., et al., *Plasma 8-epi-PGF2 alpha levels are elevated in individuals with non-insulin dependent diabetes mellitus.* FEBS Lett, 1995. **368**(2): p. 225-9.

52. Wang, B., et al., *F2-Isoprostanes Are Associated With Increased Fracture Risk in Type 2 Diabetes.* J Clin Endocrinol Metab, 2024. **00**: p. 1-8.

53. Lazutka, J.R., et al., *Effects of Short-Term Treatment with alpha-Lipoic Acid on Neuropathic Pain and Biomarkers of DNA Damage in Patients with Diabetes Mellitus.* Pharmaceuticals (Basel), 2024. **17**(11): p. 1538.

54. Sterne, J.A.C., et al., *RoB 2: a revised tool for assessing risk of bias in randomised trials.* BMJ, 2019. **366**: p. l4898.

55. Tsai, C.L., et al., *Effects of long-term Tai Chi vs. aerobic exercise on antioxidant activity and cognitive function in individuals with Parkinson's disease.* Behav Brain Res, 2025. **476**: p. 115274.
